# Digital Cognitive Behavioral Therapy for Chronic Insomnia in South Korea: Cost-Effectiveness Analysis Using Decision Tree and Markov Modeling Based on a Secondary Analysis of a Randomized Clinical Trial

**DOI:** 10.2196/71750

**Published:** 2026-01-19

**Authors:** Jung Hyun Kim, Minjeong Sohn, Jimin Woo, Jaeyong Shin, Euna Han

**Affiliations:** 1 Division of Tourism and Wellness Hankuk University of Foreign Studies Yongin Republic of Korea; 2 College of Pharmacy Yonsei Institute of Pharmaceutical Sciences, Yonsei Institute for Digital Health Yonsei University Incheon Republic of Korea; 3 AIMNEXT Inc Seoul Republic of Korea; 4 College of Medicine Yonsei University Seoul Republic of Korea

**Keywords:** insomnia, digital therapeutics, cognitive behavioral therapy, cost-effectiveness analysis, QALYs, quality-adjusted life years

## Abstract

**Background:**

Insomnia is a prevalent sleep disorder characterized by difficulty initiating or maintaining sleep and is associated with substantial health and economic burdens. Although cognitive behavioral therapy (CBT) is recommended as the first-line treatment, pharmacotherapy remains widely used despite adverse effects and significant indirect costs related to impaired productivity and workplace safety. Digital therapeutics delivering CBT through mobile platforms have emerged as scalable alternatives to improve access and outcomes. Somzz is a commercially available, domestically developed digital therapeutic that delivers CBT-based interventions for insomnia via a mobile app.

**Objective:**

This study evaluated the cost-effectiveness of Somzz compared with conventional insomnia treatment combining CBT and pharmacotherapy from both health care system and societal perspectives in South Korea.

**Methods:**

A decision-analytic model integrating a short-term decision tree with a Markov model was developed to compare costs and outcomes of digital CBT via Somzz versus conventional care in 2023. The model simulated a 27-week time horizon (three treatment cycles) and applied an annual discount rate of 4.5%. Clinical inputs, including remission probabilities and health utility values, were derived from a published randomized clinical trial comparing digital CBT delivered via Somzz with sleep hygiene education. Additional inputs, including health care resource use and unit costs, were obtained from published literature and national sources. Health outcomes were measured in quality-adjusted life years (QALYs). The cost analysis included direct medical costs and indirect costs related to absenteeism, productivity loss, and workplace accidents attributable to insomnia. Incremental cost-effectiveness ratios (ICERs) were estimated in 2023 South Korean Won (KRW). Deterministic one-way and probabilistic sensitivity analyses were conducted to assess uncertainty.

**Results:**

From a health care system perspective, digital CBT via Somzz resulted in modestly higher costs and improved health outcomes compared with standard care. Over approximately 6.5 months, Somzz generated an additional 0.0092 QALYs per patient at an incremental cost of KRW 79,691 (US $61.87), yielding an ICER of KRW 8,719,727 (US $990,883) per QALY gained. This estimate was well below the Korean willingness-to-pay threshold of KRW 30,000,000 (US $23,192.91) per QALY. From a societal perspective, digital CBT was cost-saving, producing a negative ICER due to reductions in health care utilization, workplace accidents, and productivity losses associated with higher remission rates. Sensitivity analyses identified intervention costs and remission probabilities as key drivers; however, digital CBT remained cost-effective across all scenarios under willingness-to-pay thresholds of KRW 30,000,000 and KRW 15,000,000 per QALY.

**Conclusions:**

Digital CBT for insomnia offers favorable clinical and economic value in South Korea. Using Korean clinical trial data and locally relevant societal cost inputs, this study provides policy-relevant evidence supporting early integration of digital CBT into routine insomnia care, employer health strategies, and national digital health policy.

**Trial Registration:**

World Health Organization International Clinical Trials Registry Platform KCT0007292; https://trialsearch.who.int/Trial2.aspx?TrialID=KCT0007292

## Introduction

### Problem

Insomnia is a prevalent sleep disorder among adults, characterized by difficulty initiating or maintaining sleep or early morning awakening despite adequate opportunity for sleep, resulting in poor sleep quality and daytime dysfunction [1-3]. Evidence suggests that the global prevalence of insomnia has increased, particularly during the COVID-19 pandemic, with marked rises among health care workers and individuals infected with COVID-19 [4,5]. Insomnia is associated with numerous adverse health outcomes, including a higher risk of psychiatric disorders and diminished health-related quality of life [6-8]. Furthermore, insomnia may lead to decreased work productivity and a higher risk of motor vehicle accidents [9,10]. Consequently, insomnia imposes substantial health and economic burdens, including direct costs related to health care use and medication use, as well as indirect costs from absenteeism and reduced workplace productivity [6,11].

Several treatment guidelines recommend cognitive behavioral therapy (CBT) as a first-line treatment for chronic insomnia [2,12-14]. Pharmacotherapy-centered management, despite its widespread use, is associated with adverse events, limited long-term effects, and continued health care use, while often failing to restore daily functioning and work performance [15,16]. Nevertheless, pharmacotherapy remains the most commonly used treatment in the United States and other countries, including Korea [17-19]. A survey of Korean physicians reported that pharmacotherapy was chosen by 57.14% of patients, followed by sleep hygiene education (37.11%), whereas only 1.18% received CBT [17]. This discrepancy persists despite strong evidence that CBT provides durable benefits that can be maintained for more than 1 year after treatment completion, in contrast to the short-term symptom control typically observed with pharmacotherapy [20]. As a result, indirect societal costs, such as productivity loss and accident-related costs, frequently exceed direct medical expenditures in insomnia [6,21,22].

### Review of Relevant Scholarship

Digital cognitive behavioral therapy for insomnia (dCBT-I) has emerged as a scalable solution that can overcome access barriers through the widespread availability of smartphones, tablets, and computers. Recent randomized controlled trials have demonstrated that dCBT-I not only reduces insomnia symptoms but also improves functional health, psychological well-being, and sleep-related quality of life over time [23]. Digital platforms can allow CBT to reach a wide population [24-26]. Meta-analytic evidence robustly supports the efficacy and broader benefits of digital CBT for insomnia. A 2022 meta-analysis demonstrated large short-term improvements in insomnia severity and moderate reductions in depressive symptoms that persisted long-term [27], while another meta-analysis focusing on occupational outcomes found significant decreases in lost productivity (presenteeism) and work-related rumination among working adults [28].

Building on this clinical evidence, recent economic evaluations have also matured: a 2023 systematic review and meta-analysis reported that dCBT-I consistently achieves favorable cost-utility and cost-effectiveness, particularly when societal costs such as productivity losses are considered [29]. Complementary findings from randomized controlled trials show that guided internet-delivered CBT-I can yield measurable quality-adjusted life years (QALY) gains and, from a societal perspective, has a high probability of dominating control conditions [30]. In addition, simulation modeling using a Markov framework in the United States found that fully automated digital CBT programs (eg, Sleepio) may not only be cost-effective but potentially cost-beneficial compared with no treatment, clinician-delivered CBT, and pharmacotherapy [31]. However, to date, no cost-effectiveness analysis of dCBT-I has been conducted using Korean clinical trial data and Korean-specific cost inputs. Given the strong influence of country-specific health care system structures and current treatment patterns on economic outcomes in insomnia, reliance on evidence generated in Western settings with limited consideration of societal costs may substantially misrepresent the value of digital CBT-I in the Korean context [29-32]. This study addresses an important evidence gap by incorporating Korean randomized clinical trial data and locally relevant cost inputs to evaluate the cost-effectiveness of a domestically developed digital CBT-I from both health care system and societal perspectives.

### Hypothesis, Aims, and Objectives

Somzz (AIMNEXT Inc) is the first domestically developed digital therapeutic in Korea designed to deliver CBT for insomnia (CBT-I) [33]. The fully automated mobile app incorporates core CBT-I components, including stimulus control, sleep restriction, sleep hygiene education, relaxation training, cognitive therapy, and relapse prevention, while providing personalized, real-time feedback based on users’ sleep diary data and behavioral patterns. By targeting the psychological, behavioral, and cognitive factors that maintain chronic insomnia, Somzz offers a scalable alternative to clinician-delivered CBT-I.

Based on its demonstrated clinical efficacy and the substantial societal burden of untreated insomnia, we hypothesized that digital CBT-I delivered through Somzz would represent a cost-effective strategy compared with conventional care involving CBT and pharmacotherapy. In particular, we anticipated that Somzz would improve health outcomes while reducing overall costs when broader societal impacts, such as productivity losses and accident-related costs, are taken into account.

The analysis aimed to evaluate the cost-effectiveness of Somzz compared to standard care from both health care system and societal perspectives by using clinical outcomes from a Korean randomized controlled trial (RCT) and integrating real-world Korean wage, service use, and epidemiological data. We also aimed to examine the robustness of these findings through deterministic and probabilistic sensitivity analyses.

## Methods

### Conditions and Design

**Figure 1:**
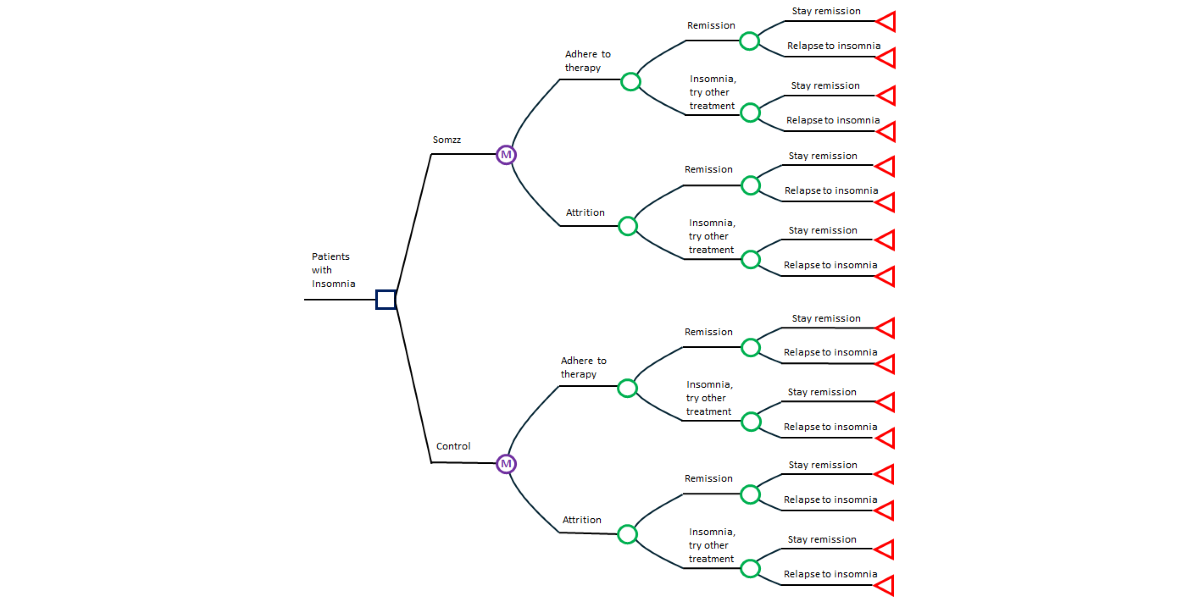
A basic description of the structure of the decision analytic Markov model.

This study is a secondary analysis of a parent single-blind RCT (trial registration: KCT0007292; registered retrospectively on 2022-05-17) to assess the safety and efficacy of a digital therapeutic, Somzz [33]. This study is to compare the costs and outcomes of Somzz versus conventional care in 2023, and thus, we constructed a static decision tree model combined with a Markov model with a 27-week (3 treatment cycles over 9 weeks), which aligns with previous insomnia cost-effectiveness studies [31]. A Markov model represents disease progression by simulating transitions between mutually exclusive health states over discrete time cycles, assuming that future transitions depend only on the current state and not the path taken to reach it [34]. In our model, patients transitioned through health states of adherence, attrition, remission, relapse, and persistence of insomnia (Figure 1).

We adopted the health care system and societal perspectives according to the economic evaluation guidelines in Korea [35]. This dual approach allowed us to assess the economic implications of a health care intervention more thoroughly by capturing the direct financial impact on the health care system and broader economic and societal effects. A discount of 4.5% was applied as per Korean guidelines [35]. We referred to the Consolidated Health Economic Evaluation Reporting Standards (CHEERS) guidelines [36] to conduct the overall cost-effectiveness analysis (Supplementary Table S1 in Multimedia Appendix 1).

### Inclusion and Exclusion Criteria

In the aforementioned parent trial, participants were eligible if they were (1) ≥19 years old, (2) had a diagnosis of chronic insomnia, and (3) were able to participate in scheduled visits and assessments. Exclusion criteria in the original trial included: (1) severe psychiatric or neurological conditions, (2) participation in other sleep-related interventions, and (3) medical contraindications to CBT-I or study procedures [33]. This study included only participants from the original trial dataset; no additional inclusion or exclusion criteria were applied.

### Participant Characteristics

The parent RCT enrolled 98 adults (Somzz: 49; control: 49). Key characteristics were (1) age: adults 19 years and older (details reported in original trial [33]), (2) employment: 51% engaged in paid work (Somzz: 22/49; control: 28/49), and (3) baseline insomnia severity: clinically significant chronic insomnia. Participants were similar between groups due to randomization [33].

### Sampling Procedures

In the aforementioned parental randomized controlled trial, participants were recruited through multiple clinical centers via advertisements and clinician referrals [33]. Sampling followed consecutive enrollment of eligible patients [33]. The sample for this cost-effectiveness study comprises all randomized participants with complete outcome data in the parental trial [33].

### Sample Size, Power, and Precision

The sample size was determined in the parent clinical trial based on detecting meaningful differences in insomnia severity (Insomnia Severity Index [ISI] scores) [33]. For the current economic evaluation, all available trial participants were included; therefore, no additional power analysis was required. Precision was improved through probabilistic sensitivity analyses using distributions for all parameters.

### Measures and Covariates

#### Intervention and Comparator

The intervention is the digital CBT, Somzz, for insomnia treatment, and the comparator is CBT or pharmacotherapy without Somzz. As aforementioned, underpinning this study was a multicenter, single-blind, RCT (trial registration: KCT0007292) to assess the safety and efficacy of Somzz [33]. This study evaluates the cost-effectiveness of the Somzz using the collected and published information from the aforementioned parent clinical trial [33].

#### Outcomes

Outcomes included direct efficacy indicators related to insomnia treatment indicators (probabilities of remission, maintaining remission, attrition) and quality-adjusted-life-years. The direct efficacy values were extracted from the parent clinical trial [33]; (1) remission was defined as an ISI score of 7 or lower, indicating the absence of clinically significant insomnia; (2) the attrition; and (3) the probability of maintaining remission, which were measured during the follow-up at 18 weeks after device withdrawal. The minimum value of the attrition rate was the lower limit of the 95% CI, assuming that the attrition rate followed a normal distribution in the population. The maximum value was determined by applying values obtained from the literature [37], considering the possibility of an underestimated dropout rate in the clinical trial. QALYs were used as a health outcome measure in the analysis. QALYs were derived from the parent clinical trial [33], which measured QALY with the Short Form 6 Dimensions (SF-6D) Health State Short Form (a subset of 6 items from the Short Form Health Survey Version 1) [38]. The SF-6D includes 6 dimensions, each with 2 to 5 levels, and is capable of generating 7500 different health states. Brazier algorithm was used to calculate utility values [39]. The SF-6D was reported to show superior sensitivity to changes in mild-to-moderate physical and mental health conditions compared to the EQ-5D-3L [40,41].

#### Direct Medical Costs

We defined direct medical costs related to insomnia as expenses incurred from visits to clinics, hospitals, or pharmacies, specifically for insomnia treatment. The base case value for the treatment group was determined as the sum of 2 consultation fees, the cost of CBT software, and medication expenses. The minimum value for the treatment group was calculated as the 2 consultation fees, assuming no costs for CBT software or medication. The upper limit value for the treatment group was calculated by increasing the CBT software cost up to 200,000 KRW (US $155.28) with 2 consultations and related medications. The primary value for the control group included only medication costs, which were adjusted for adherence rates of 14% for prescription medications and 2% for over-the-counter medications. The minimum value of medication costs was calculated as the mean prescription medication cost subtracted from the SD. The maximum value was derived by adding the SD to the mean prescription medication cost. Medical expenses unrelated to insomnia treatment were classified as indirect medical costs.

#### Indirect Costs

Indirect costs were calculated as the sum of absenteeism, productivity loss, and workplace accident costs. Absenteeism and productivity losses caused by insomnia were monetized as part of absenteeism costs. We converted the number of days absent from work (cost of absenteeism) and the degree of productivity decline (cost of reduced work productivity) due to insomnia into a monetary cost for wage workers participating in the clinical trial [33]. Insomnia increases the likelihood of workplace accidents; therefore, the associated costs were estimated by multiplying the probability of insomnia-related accidents by the average cost per workplace accident. Insomnia can cause workplace accidents; therefore, we calculated the cost of insomnia-related accidents by multiplying the probability of an accident occurring because of insomnia by the average cost of an accident.

### Data Collection

All clinical and quality-of-life data were collected prospectively during the parent RCT at 6 scheduled visits (screening, baseline, 1 week, 2 weeks, 4 weeks, and 18 weeks) [33]. Economic variables were derived from Korean national databases, trial-reported work impairment, and published literature. All data were de-identified before secondary analysis.

### Quality of Measurements

Clinical outcomes (eg, ISI) were collected in the parent clinical trial by trained personnel following standardized protocols [33]. Economic parameters used validated national datasets. No missing data were reported in the parent clinical trial; no new missingness occurred in the secondary dataset.

### Instrumentation

The instrumentation for this study included the Somzz dCBT-I mobile app, which delivers standardized CBT modules for insomnia through a smartphone-based digital platform, and the SF-6D survey instrument, a validated and widely used tool for measuring health-related utility values. All instruments were used in their original, validated formats, and no modifications were made to any components or measures.

### Masking

The parent clinical trial used a single-blind design in which outcome assessors were blinded to treatment assignment. Participants were aware of their intervention due to the nature of the digital therapeutic. The economic evaluation was fully blinded to group identifiers [33].

### Psychometrics

The SF-6D utilities were derived from SF-36 responses, and the psychometric properties of the Korean SF-36 support its use in Korean populations. In a Korean general population study (N=600), the SF-36 demonstrated acceptable internal consistency and test–retest reliability, with reliability coefficients ranging from 0.54 to 0.80 across domains [38,39,42]. In addition, the Korean Health Insurance Review and Assessment Service economic evaluation guideline allows the use of multi-attribute utility instruments, including SF-6D–derived utilities, in the Korean health technology assessment context [35]. Utility scoring used validated country-neutral algorithms. Attrition and remission measurements followed established ISI-based definitions [33].

### Data Diagnostics

We evaluated plausibility ranges for all parameters, checked for outliers in cost distributions, and verified internal model consistency. The attrition rate distribution was modeled using normal assumptions based on trial data. Costs from claims databases were cross-validated with external Korean sources.

### Analytic Strategy

#### Base-Case Analysis

The base-case analysis estimated the incremental cost-effectiveness ratio (ICER) by comparing the costs and health outcomes of digital CBT with those of treatment as usual. Health outcomes were measured in QALYs, and the analysis incorporated both direct medical costs and indirect societal costs to comprehensively assess the economic impact of the intervention.

#### Sensitivity Analysis

We ensured the robustness of our findings by varying several key assumptions, including the frequency of clinic visits, the need for additional consultations and educational sessions, and incremental health care expenditures related to insomnia. We also varied the financial impact of absenteeism, presenteeism, and workplace accidents resulting from insomnia. For absenteeism and presenteeism, we alternatively used data from the Work Productivity and Activity Impairment, which provides average, maximum, and minimum values for these factors [43]. We established the minimum cost of workplace accidents as zero with a scenario of no accident occurrence and the maximum cost as the highest individual accident cost recorded in the Industrial Accident Insurance Statistics [44] to account for the most severe financial impact observed.

### Ethical Considerations

The trial was approved by the institutional review boards (IRBs) of all participating centers and conducted in accordance with the Declaration of Helsinki and Good Clinical Practice guidelines [33]. This study is a secondary analysis using existing data with primary consent, and the original consent or IRB approval covers secondary analysis without additional consent. Furthermore, ethical approval for the cost-effectiveness study was obtained from the Institutional Review Board of Yonsei University (approval number 202111-HR-2636-01). A waiver of informed consent for the use of retrospective data was also granted by the Institutional Review Board of Yonsei University. No financial incentives were provided in the parental clinical trial [33]. All data were deidentified before analysis. This study involved secondary analysis of anonymized clinical trial data already covered under the original IRB approval [33]. No images or supplementary materials in this article contain identifiable information about individual participants or users. All figures and tables present aggregated or schematic data only. Therefore, no additional consent for the use of identifiable images was required. If any materials containing identifiable information were to be included in the future, we would obtain explicit written consent from those individuals and upload the corresponding consent documentation in accordance with journal guidelines.

## Results

### Participant Flow

#### Overview

In the parental clinical trial, a total of 98 adults with chronic insomnia were enrolled and randomized in a 1:1 ratio to the Somzz group (n=49) or the control (sleep hygiene education [SHE]) group (n=49). All participants initiated the assigned intervention, and all participants had 6 scheduled visits (screening, baseline, 1 week, 2 weeks, 4 weeks, and 18 weeks as post intervention) over 6-9 months. The attrition rate in the Somzz group was 12% (6/49), whereas the SHE group showed comparable retention (5/49, 10%) over the 4-month follow-up period. Outcome analyses were conducted according to the intention-to-treat principle [33].

#### Recruitment

Participants were recruited from 3 university-affiliated hospitals through outpatient clinics and study advertisements. Eligible individuals were screened by research staff and invited to participate between baseline and randomization. After informed consent, participants completed baseline assessments and were randomly assigned to the Somzz or SHE condition using a single-blind allocation procedure [33].

#### Baseline Characteristics

At baseline, the 2 groups were comparable in demographic and clinical characteristics. Participants were adults with chronic insomnia and demonstrated similar insomnia severity, sleep diary metrics, and mental health profiles prior to randomization. No meaningful group differences were observed at baseline, supporting the validity of the randomization process [33].

#### Primary Outcomes

Outcomes included direct efficacy indicators related to insomnia treatment indicators (probabilities of remission, maintaining remission, and attrition) and quality-adjusted-life-years. The direct efficacy values were extracted from the parent clinical trial [33]; (1) remission was 51% (22/43) of the digital CBT group and 14% (6/44) of the control group post intervention; (2) the attrition rate was 12% (6/49) for the in the digital CBT group and 10% (5/49) in the control group; (3) the probability of maintaining remission was 35% (15/43) of the treatment group and 11% (5/44) of the control group. For indirect medical costs, we compared medical claims from individuals diagnosed with insomnia to those without an insomnia diagnosis and determined that excess health expenditure caused by insomnia would amount to 553,625 KRW (US $429.83) over 9 weeks (Table 1).

Indirect costs were calculated as the sum of absenteeism, productivity loss, and workplace accident costs. In a parent clinical study, 54% were “individuals employed in paid work,” and they reported being absent from work because of insomnia-related issues for 3.11 hours per week on average before starting treatment [33]. Therefore, we calculated the cost of absenteeism due to insomnia per one 9-week treatment cycle as follows: 9 weeks × 3.11h/week × 19,806 KRW (US $15.38) (hourly wage) × 54% (employment rate) ≒ 33,312 KRW (US $25.87). The parent clinical trial participants reported a 55% decline in productivity caused by insomnia [33]. Therefore, the indirect cost of lost productivity for one 9-week treatment cycle was approximated as follows: 45 days (5 days of work per week for the 9-week treatment period) × 55% (productivity loss due to insomnia) × 157,260 KRW (US $122.10)/day (daily wage) × 54% (employment rate) ≒ 2,101,347 KRW (US $1631.49) (Table 1). The probability of an insomnia-related workplace accident was assumed to be 1% [11]. According to the Ministry of Employment and Labor’s Industrial Accident Insurance Statistics in Korea, the total amount of industrial accident insurance benefits in 2020 was 6.00 trillion KRW paid across 350,000 beneficiaries, resulting in an average of 17 million KRW per person [44]. Thus, the estimated accident cost per person is 85 million KRW annually and approximately 79,562 KRW (US $61.77) for a 9-week period [45,46] (Table 1).

**Table 1:** Model input parameters for the cost-effectiveness analysis of digital CBT (Somzz) for chronic insomnia among Korean adults.

Parameters^a^				Value assumed (range)		Distribution		Source
**Outcomes**								
	**Remission probability**							
		Treatment group (Somzz)	0.51 (0.40-0.68)		Beta		[33], ISI^b^<7; (minimum ISI < 6 - maximum ISI < 8)	
		Control group	0.14 (0.09-0.40)		Beta		[33]	
	**Probability of maintaining remission for 18 weeks**							
		Treatment group (Somzz)	0.35 (0.18-0.47)		Beta		[33] (minimum ISI<6 - maximum ISI<8)	
		Control group	0.11 (0.09-0.18)		Beta		[33]	
	**Attrition rate**							
		Treatment group (Somzz)	0.12 (0.08-0.30)		Beta		[33]; (minimum lower level of 95% CI- maximum [37])	
		Control group	0.10 (0.06-0.40)		Beta		[33]; (minimum [33] - maximum [31])	
	**Quality-adjusted life years**							
		Insomnia	0.684 (0.460-1.0)		Beta		[33]	
		Noninsomnia	0.810 (0.573-1.0)		Beta		[33]	
**Costs**								
	**Direct medical costs related to insomnia**							
		Treatment group (Somzz)	KRW 91,194 (29,100-237,194)^c^		Gamma		[47,48]	
		Control group	KRW 8,094 (7,890-10,068)^d^		Gamma		[47,48]	
	**Direct medical costs unrelated to insomnia**							
		Treatment group (Somzz)	KRW 553,625 (0-136,074,635)^e^		gamma		[49]	
		Control group	KRW 437,689 (0-89,272,731)^f^		Gamma		[49]	
	**Indirect costs**							
		Workplace absenteeism	KRW 33,312 (0-278,497)^g^		Gamma		[33], [45,46]	
		Workplace presenteeism	KRW 2,101,347 (0-3,827,195)^h^		Gamma		[33], [45,46]	
		Additional costs from workplace accidents	KRW 79,562 (0-1,590,541)^I^		Gamma		[33], [11,45,46]	
	**Additional costs**							
		Digital CBT^j^	KRW 91,194 (29,100-237,194)^k^		Gamma		Internal data	

^a^Model input parameters for the cost-effectiveness analysis of digital cognitive behavioral therapy (Somzz) for chronic insomnia among Korean adults were derived from clinical trial data and relevant literature. These parameters included remission and attrition probabilities, health-state utilities, direct and indirect costs, and the corresponding probability distributions used for probabilistic modeling.

^b^ISI: Insomnia Severity Index.

^c^US $70.80 (US $22.59-184.16).

^d^US $6.29 (US $6.13-7.82).

^e^US $429.83 (US $0-105648.35).

^f^US $339.82 (US $0-69311.35).

^g^US $25.86 (US $0-216.23).

^h^US $1631.49 (US $0-2971.43).

^i^US $61.77 (US $0-1234.90).

^j^CBT: cognitive behavioral therapy.

^k^US $70.80 (US $22.59-184.16).

### Statistics and Data Analysis

#### Cost-Effectiveness of Digital CBT

Table 2 presents scenarios detailing the simulated health utility (QALYs) and cost outcomes for the baseline model. All scenarios, except Scenario 4, were analyzed by including only direct medical costs. Costs and QALYs increased for patients who implemented the digital CBT solution. The model predicted that the use of digital software to treat insomnia would produce 0.3701 QALYs and 101,729 KRW (US $78.98) of expected costs compared with 0.3609 QALYs and 22,038 KRW generated by treatment as usual without the aid of digital software over 6.5 months. Introducing the digital insomnia solution would increase costs by 79,691 KRW (US $61.87) and QALYs by 0.0091. The ICER associated with insomnia treatment using digital software was 8,719,727 KRW (US $6770.00) per QALY gained, which is well below the hypothetical threshold value in Korea of 30,000,000 KRW (US $23,292)/QALY gained.

**Table 2:** Cost-effectiveness analysis of digital cognitive behavioral therapy (Somzz) for chronic insomnia among Korean adults relative to the control group by base case and scenario.

Strategy			Cost, KRW (US $)		Effectiveness (QALYs)^a^		Incremental cost, KRW (US $)		Incremental effectiveness (QALYs)		ICER^b^, KRW /QALYs (US $/QALYs)
**Base case: 2 clinic visits**											
	Control group	22,038 (17.11)		0.3609		—^c^		—		—	
	Treatment group (Somzz)	101,729 (78.98)		0.3701		79,691 (61.88)		0.0091		8,719,727 (6,770.87)	
**Scenario 1: 3 clinic visits**											
	Control group	22,038 (17.11)		0.3609		—		—		—	
	Treatment group (Somzz)	113,859 (88.40)		0.3701		91,821 (71.30)		0.0091		10,046,980 (7,801.48)	
**Scenario 2: 2 clinic visits + educational and consultant fees**											
	Control group	22,038 (17.11)		0.3609		—		—		—	
	Treatment group (Somzz)	131,729 (102.27)		0.3701		109,691 (85.18)		0.0091		12,002,298 (9,319.78)	
**Scenario 3: 3 clinic visits + educational and consultant fees**											
	Control group	22,038 (17.11)		0.3609		—		—		—	
	Treatment group (Somzz)	143,859 (111.69)		0.3701		121,821 (94.59)		0.0091		13,329,551 (10,350.40)	
**Scenario 4: all costs included^d^**											
	Control group	7,679,657 (596.49)		0.3609		—		—		—	
	Treatment group (Somzz)	6,777,978 (526.42)		0.3701		–901,678 (–700.15)		0.0091		–98,660,780 (76,610.10)	

^a^QALYs: quality-adjusted life years.

^b^ICER: incremental cost-effectiveness ratio.

^c^Not available.

^d^Direct medical costs related to insomnia + (educational and consultation fees) + direct medical costs unrelated to insomnia + indirect costs.

Compared to the control group, the ICER for insomnia treatment using the digital intervention, assuming all possible indirect costs, yielded a lower cost of 901,678 KRW (US $700.06) and higher QALYs of 0.0091. This negative ICER arises from combining the reduced total costs and increased health utility. Digital CBT reduces insomnia-related indirect costs, such as health care expenses and lost workplace productivity, through higher effectiveness and remission rates. These cost reductions surpass the direct costs associated with digital CBT (Table 2).

#### Sensitivity Analysis

**Figure 2:**
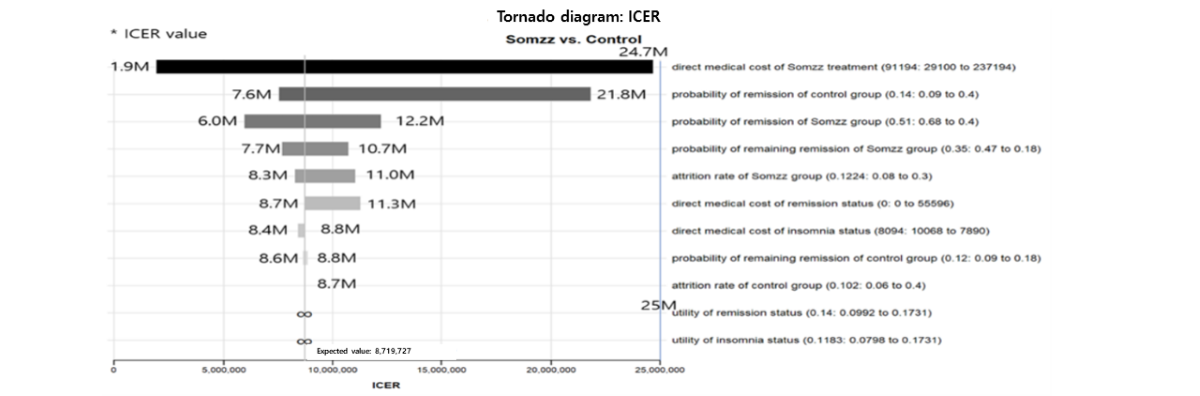
Tornado diagram for incremental cost-effectiveness ratio. ICER: incremental cost-effectiveness ratio.

**Figure 3:**
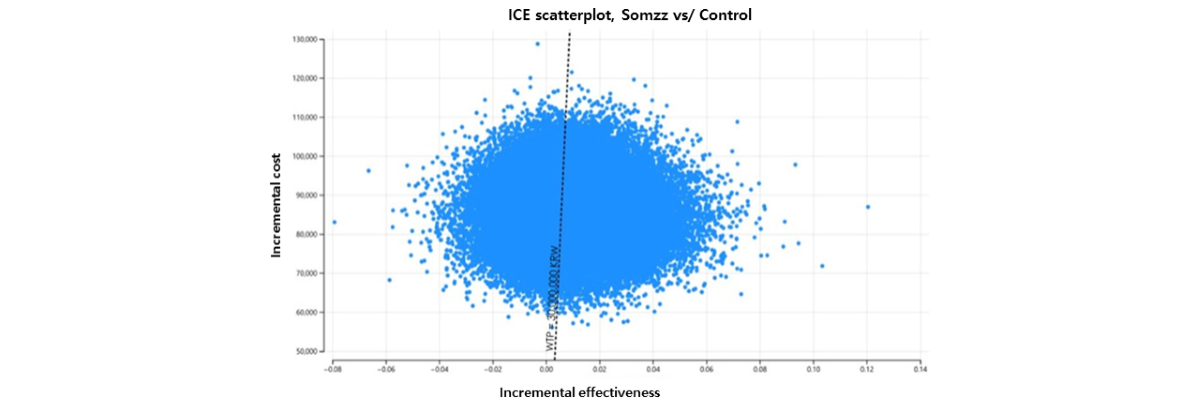
Probabilistic sensitivity analysis results: incremental cost-effectiveness scatterplot (estimates based on the 95% CI).

We conducted a one-way deterministic and probabilistic sensitivity analysis to test the robustness of our findings. Figure 2 shows a tornado diagram of the results of this analysis. The direct costs of the digital CBT group, which ranged from 29,100 to 237,194 KRW (US $22.59 to US $184.16), had the largest impact on the ICER, followed by the probability of remission in the control group. These parameters had the greatest impact in all scenarios (Multimedia Appendices 2-4). The Monte Carlo simulation results indicated that digital CBT is the optimal cost-effective strategy with a probability of 67.4%, represented as an ICE scatterplot in Figure 3.

For Scenarios 1-3, digital CBT was the most cost-effective strategy, with probabilities of 66.9%, 65.1%, and 63.9%, respectively (Multimedia Appendices 5-7). Furthermore, digital CBT was identified as the most cost-effective strategy at a willingness-to-pay threshold of 15,000,000 KRW (US $11,647.5), with probabilities of 58.8%, 57.8%, 53.5%, and 51.3% for the base case and Scenarios 1-3, respectively (Multimedia Appendix 8).

## Discussion

### Support of Original Hypotheses

We combined a decision tree and the Markov model to assess the cost-effectiveness of digital CBT for adults seeking treatment for insomnia in South Korea. While several prior studies have evaluated the cost-effectiveness of digital CBT for insomnia, most were conducted in Western health care systems [29-32]. In contrast, this study evaluates a regulated, commercially available digital therapeutic using Korean randomized trial data and explicitly incorporates societal costs that are highly relevant to the Korean labor and health care context. From a health care perspective, digital CBT slightly increased health care costs while improving health outcomes compared with standard care. In contrast, from a societal perspective, digital CBT was cost-beneficial, yielding a negative ICER. Accordingly, the digital therapeutic evaluated in this study was highly cost-effective for functional outcomes in insomnia treatment at a willingness-to-pay threshold of 30,000,000 KRW (US $23,295) per QALY gained and remained cost-effective at a lower threshold of 15,000,000 KRW (US $11,647.5) per QALY gained. These findings underscore that incorporating broader societal costs meaningfully changes the economic value of insomnia treatment interventions. Reduced health care use, lower risks of workplace accidents, and improved work productivity associated with insomnia remission were key drivers of cost-effectiveness. Both deterministic and probabilistic sensitivity analyses confirmed the robustness of these results.

### Similarity of Results and Interpretation

These results align with those of previous studies that found guided digital CBT to be cost-effective [30-32]. One strength of our study was the inclusion of indirect costs. We considered reductions in health care expenditure as effective insomnia treatment can lead to fewer medical visits, less need for medication, and lower overall health care use [21,22]. Furthermore, we accounted for a decreased risk of workplace accidents. Insomnia impairs cognitive and physical functioning, which increases the likelihood of errors and accidents at work [50,51]. Treating insomnia can mitigate this risk, thereby leading to safer work environments. Our analysis also included improved workplace productivity. Insomnia negatively affects concentration, efficiency, and overall job performance [52,53]. Therefore, effective treatment can enhance productivity by improving employees’ sleep quality and daytime functioning. By capturing these indirect costs, our analysis demonstrates the broader economic benefits of insomnia treatment beyond immediate improvements in individual health. Insomnia is linked to 7.2% of all costly workplace accidents and errors, and accounts for 23.7% of the total costs associated with these incidents [11]. Not including these additional costs could underestimate the total economic burden of insomnia and potential costs saved through the application of effective treatment. In contrast, including costs related to workplace accidents and errors could reveal higher overall costs, and thus, a greater potential for cost savings through insomnia treatment.

The demand for effective insomnia treatment is substantial, with 20%-30% of the population experiencing insomnia symptoms [1-3]. However, several factors hinder traditional clinician-delivered CBT, including the availability of trained therapists and the need for patients to attend scheduled sessions, which many people can find challenging. Patients often face difficulties in taking time off work and securing childcare or older adult care to attend in-person therapy sessions. These logistical challenges limit the reach and effectiveness of clinician-delivered CBT [54].

Digital CBT delivery offers a promising solution to these accessibility issues by enabling instant and unlimited access to CBT resources, thereby allowing patients to engage with therapy at any time and from any location, effectively eliminating waiting times. This flexibility is crucial for patients who may be unable to attend regular appointments because of their busy schedules [54]. Digital platforms can make CBT more widely available, ensuring that more individuals who require treatment can access it promptly and conveniently. This approach not only expands the reach of effective insomnia treatment but also aligns with the high demand for such interventions, ultimately helping bridge the gap between the recommended use of CBT and its actual accessibility and use.

### Generalizability

The generalizability of this study’s findings should be interpreted in the context of the Korean population and health care system. Participants were digitally literate adults recruited from university hospitals [33], which may not fully represent individuals with limited technology access or older adults who may engage differently with mobile health interventions. Nonetheless, given Korea’s high smartphone penetration rate and increasing familiarity with digital health tools, Somzz has strong potential for scalable implementation across the broader adult population.

Economic estimates in this model reflect Korean wage levels, health care use patterns, and societal cost structures. Although absolute cost differences may vary in other countries, the primary mechanisms driving cost-effectiveness, such as higher remission rates, reduced health care use, and improved productivity, are likely applicable to other high-income settings with similar burdens of insomnia and labor market characteristics. Because Somzz was developed within the Korean cultural and linguistic environment, differences in cultural attitudes toward sleep, mental health, and digital therapeutics may influence usability and adherence in other populations. Additionally, real-world engagement with digital CBT-I may vary outside a controlled trial setting, which could influence long-term effectiveness and economic outcomes.

Overall, while these findings are most directly applicable to technologically advanced health care systems with strong digital readiness, they provide valuable insights for other countries considering digital CBT-I adoption. Extrapolation should be undertaken cautiously with attention to cultural, economic, and infrastructural differences.

### Implications

This study provides novel evidence by incorporating indirect societal costs, including productivity loss and workplace accidents, into the economic evaluation of digital CBT, dimensions that are rarely captured in prior analyses. Importantly, by focusing on a digital therapeutic that meets regulatory standards and is designed for real-world implementation, this study extends prior digital CBT economic evaluations toward policy-relevant evidence for reimbursable digital therapeutics. By using Korean clinical trial data [33] and locally relevant economic parameters rather than relying on Western-based assumptions, the study offers population-specific insights that more accurately reflect real-world conditions. This comprehensive approach strengthens the relevance of our findings for clinicians, policymakers, and employers by demonstrating the broader economic and workplace impact of digital CBT and informing decision-making for large-scale adoption within the Korean health care and occupational health systems.

One limitation of this study was the relatively short follow-up period. Although a period of 6.5 months is approximately the same or longer than that used in all previous studies on the cost-effectiveness of CBT for insomnia, it may not be sufficient to fully capture the long-term economic benefits of treatment. Simulating costs over a longer period, such as 12 or 24 months, could more effectively demonstrate potential long-term cost reductions for employers. A longer time horizon may allow for a more significant accumulation of treatment benefits, suggesting that our current results may be conservative.

Another limitation is the sample size. Economic evaluations conducted in parallel with clinical trials often face power issues because cost variables typically have a higher variance and require larger sample sizes than clinical outcomes to achieve statistical significance. Considering these challenges, we adopted a probabilistic decision-making approach to account for the uncertainty and variability in cost and utility estimates. This approach helped us perform a more robust analysis despite the limitations of the sample size and follow-up duration in this study.

Finally, the parent clinical trial from which outcome data were extracted [33] was not structured to compare traditional and digital CBT directly, primarily because of practical constraints (eg, despite having insomnia symptoms, only a small proportion of patients actively seek pharmacotherapy in the real world). This approach mimics real-world scenarios in which patients often receive a combination of treatments rather than a single isolated therapy.

This study did not involve direct patient or stakeholder engagement. However, the societal burden and impact of insomnia were incorporated into the analytical framework. No direct involvement from patients, service recipients, the general public, or other stakeholders influenced the approach or findings of this study.

### Conclusions

The results of this study suggest the significant potential of early integration of digital CBT into insomnia treatment to improve health care outcomes and generate substantial cost-effectiveness. These findings underscore the importance of incorporating such technology as a valuable enhancement to routine medical practice, thereby ultimately enhancing overall insomnia care. Beyond demonstrating cost-effectiveness, this study contributes to the growing evidence base supporting digital therapeutics by illustrating how locally grounded economic evaluations can inform reimbursement, employer adoption, and national digital health policy. Given these promising results, future research should focus on expanding access to digital CBT and assessing its effectiveness on a population-wide scale. Understanding the effectiveness of digital CBT in real-world settings, in which factors such as varying levels of access, patient engagement, and implementation challenges can influence outcomes, is crucial.

## Appendix

Multimedia Appendix 1 Supplementary Table 1.

Multimedia Appendix 2 Deterministic sensitivity analysis results: Scenario 1 – tornado diagram for ICER.

Multimedia Appendix 3 Deterministic sensitivity analysis results: Scenario 2 – tornado diagram for ICER.

Multimedia Appendix 4 Probabilistic sensitivity analysis results: incremental cost-effectiveness scatterplot for Scenario 3.

Multimedia Appendix 5 Probabilistic sensitivity analysis results: incremental cost-effectiveness scatterplot for Scenario 1.

Multimedia Appendix 6 Probabilistic sensitivity analysis results: incremental cost-effectiveness scatterplot for Scenario 2.

Multimedia Appendix 7 Probabilistic sensitivity analysis results: incremental cost-effectiveness scatterplot for Scenario 3.

Multimedia Appendix 8 Probabilistic sensitivity analysis results: incremental cost-effectiveness scatterplot: (A) base case; (B) Scenario 1; (C) Scenario 2; and (D) Scenario 3 (WTP = 15,000,000).

## Abbreviations

CBT: cognitive behavioral therapy
CHEERS: Consolidated Health Economic Evaluation Reporting Standards
dCBT-I: digital cognitive behavioral therapy for insomnia
ICER: incremental cost-effectiveness ratio
IRB: institutional review board
ISI: Insomnia Severity Index
QALY: quality-adjusted life years
RCT: randomized controlled trial
SF-6D: Short Form 6 Dimensions

## Data Availability

The datasets generated or analyzed during this study are available from the corresponding author on reasonable request. Most of the input data used to derive the results of this study are extracted from published literature, which is all listed in the Reference section with corresponding citations in the text.
