# Supplementary material for: Digital Cognitive Behavioral Therapy for Chronic Insomnia in South Korea: Cost-Effectiveness Analysis Using Decision Tree and Markov Modeling Based on a Secondary Analysis of a Randomized Clinical Trial
Source: JMIR Mhealth Uhealth. 2026 Jan 19;14:e71750. doi: 10.2196/71750 (PMC12865351; doi:10.2196/71750)
Supplement: Multimedia Appendix 1 [file mhealth_v14i1e71750_app1.pdf]

Section/topic	Item	Guidance for reporting	Reported in section
Title	1	Identify the study as an economic evaluation and specify the interventions being compared.	Title clearly states "Cost-Effectiveness Analysis"
Abstract	2	Provide a structured summary that highlights context, key methods, results, and alternative analyses.	Abstract includes all required elements
Background and objectives	3	Give the context for the study, the study question, and its practical relevance for decision making in policy or practice.	Introduction section
Health economic analysis plar	4	Indicate whether a health economic analysis plan was developed and where available.	Not registered; not mentioned in manuscript
Study population	5	Describe characteristics of the study population (such as age range, demographics, socioeconomic, or clinical characteristics).	Demographics section, page 6
Setting and location	6	Provide relevant contextual information that may influence findings.	Introduction and Methods – South Korea context
Comparators	7	Describe the interventions or strategies being compared and why chosen.	Intervention and Comparators, pages 5–6
Perspective	8	State the perspective(s) adopted by the study and why chosen.	Methods – Healthcare system and societal perspectives used
Time horizon	9	State the time horizon for the study and why appropriate.	Methods – 6.5 months stated; modeled over 27 weeks
Discount rate	10	Report the discount rate(s) and reason chosen.	Methods – 4.5% for both costs and QALYs per Korean guidelines
Selection of outcomes	11	Describe what outcomes were used as the measure(s) of benefit(s) and harm(s).	Methods – QALYs derived from SF-6D
Measurement of outcomes	12	Describe how outcomes used to capture benefit(s) and harm(s) were measured.	SF-6D used within clinical trial; Brazier algorithm applied
Valuation of outcomes	13	Describe the population and methods used to measure and value outcomes.	Utility calculation via SF-6D and Brazier method (p. 9)
Measurement and valuation o	14	Describe how costs were valued.	Pages 9–12 – Internal clinical trial and published literature
Currency, price date, and conv	15	Report the dates of the estimated resource quantities and unit costs, plus the currency and year of conversion.	All costs in KRW, adjusted to 2023 values
Rationale and description of r	16	If modelling is used, describe in detail and why used. Report if the model is publicly available and where it can be accessed.	Figure 2, Model Structure section
Analytics and assumptions	17	Describe any methods for analysing or statistically transforming data, any extrapolation methods, and approaches for validating any model used.	Deterministic and probabilistic sensitivity analyses; assumptions described throughout Methods
Characterising heterogeneity	18	Describe any methods used for estimating how the results of the **study vary for subgroups.**	Not performed; no subgroups analyzed
Characterising distributional e	19	Describe how impacts are distributed across different individuals or adjustments made to reflect priority populations.	Not analyzed; not applicable for this study
Characterising uncertainty	20	Describe methods to characterise any sources of uncertainty in the analysis.	Sensitivity analysis section, pages 14–15
Approach to engagement witl	21	Describe any approaches to engage patients or service recipients, the general public, communities, or stakeholders (such as clinicians or payers) in the design of the study.	Not addressed
Study parameters	22	Report all analytic inputs (such as values, ranges, references) including uncertainty or distributional assumptions.	Table 1 – model input parameters
Summary of main results	23	Report the mean values for the main categories of costs and outcomes of interest and summarise them in the most appropriate overall measure.	Table 2 – ICERs and incremental QALYs reported
Effect of uncertainty	24	Describe how uncertainty about analytic judgments, inputs, or projections affect findings. Report the effect of choice of discount rate and time horizon, if applicable.	Tornado diagrams, PSA, scenario analyses
Effect of engagement with pa	25	Report on any difference patient/service recipient, general public, community, or stakeholder involvement made to the approach or findings of the study	Not applicable
Study findings, limitations, ge	26	Report key findings, limitations, ethical or equity considerations not captured, and how these could affect patients, policy, or practice.	Discussion section (p. 16–18) covers context, robustness, and policy relevance
Source of funding	27	Describe how the study was funded and any role of the funder in the identification, design, conduct, and reporting of the analysis	Final page – MoHW and HUFS; no involvement stated
Conflicts of interest	28	Report authors conflicts of interest according to journal or International Committee of Medical Journal Editors requirements.	Final page – disclosed affiliations and conflicts
