# Supplementary figures and images for: Digital Cognitive Behavioral Therapy for Chronic Insomnia in South Korea: Cost-Effectiveness Analysis Using Decision Tree and Markov Modeling Based on a Secondary Analysis of a Randomized Clinical Trial
Source: JMIR Mhealth Uhealth. 2026 Jan 19;14:e71750. doi: 10.2196/71750 (PMC12865351; doi:10.2196/71750)

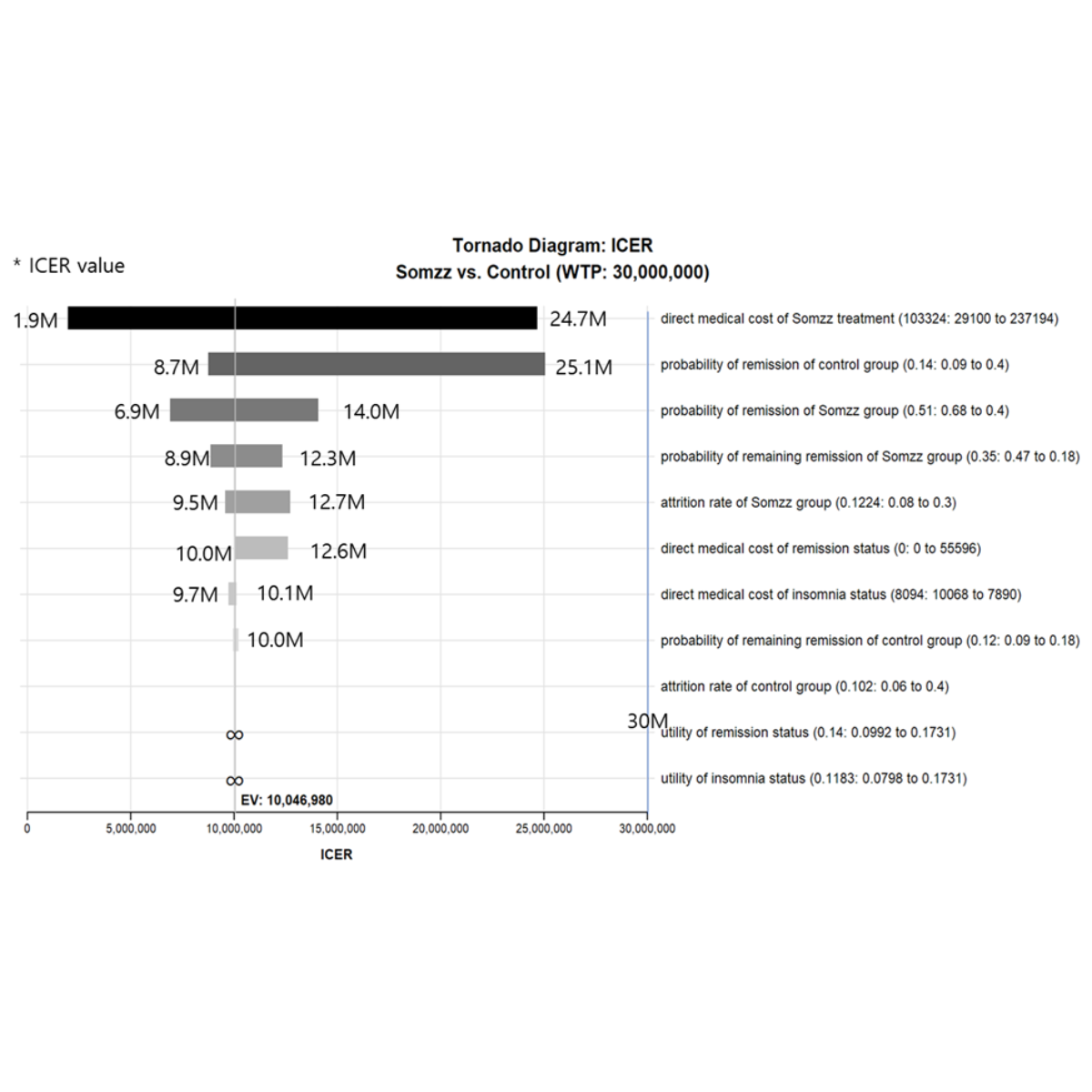

Supplement: Multimedia Appendix 2 [file mhealth_v14i1e71750_app2.png]

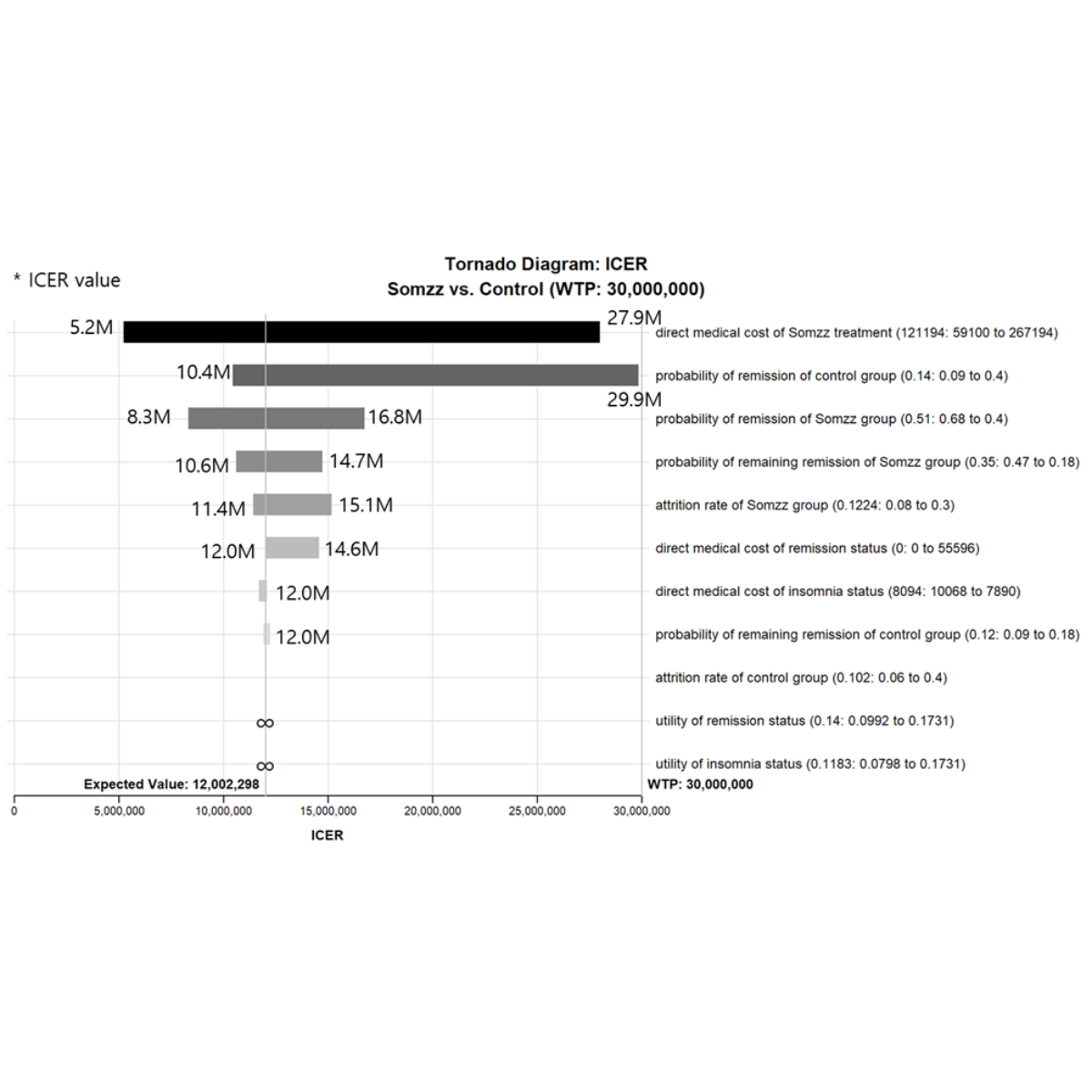

Supplement: Multimedia Appendix 3 [file mhealth_v14i1e71750_app3.png]

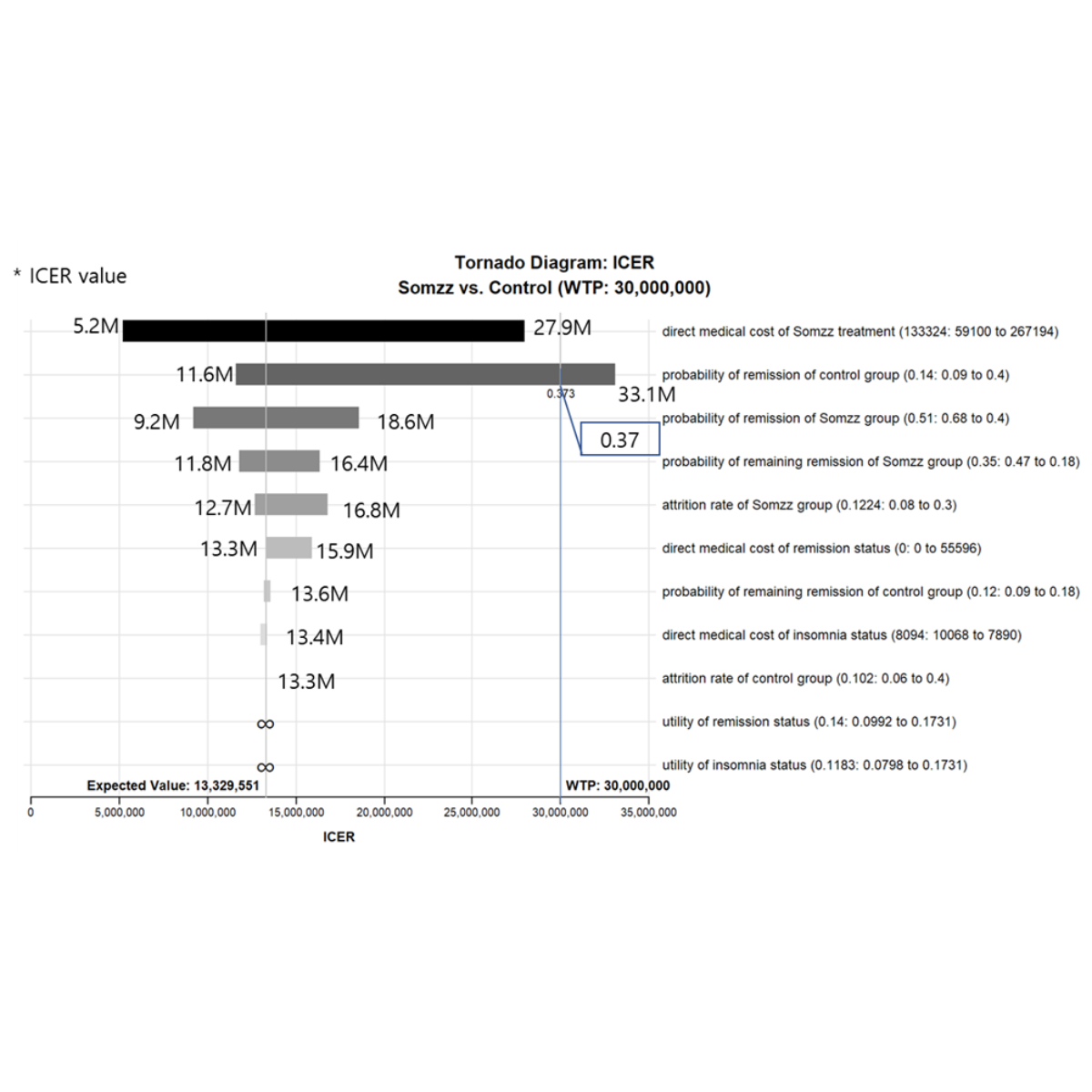

Supplement: Multimedia Appendix 4 [file mhealth_v14i1e71750_app4.png]

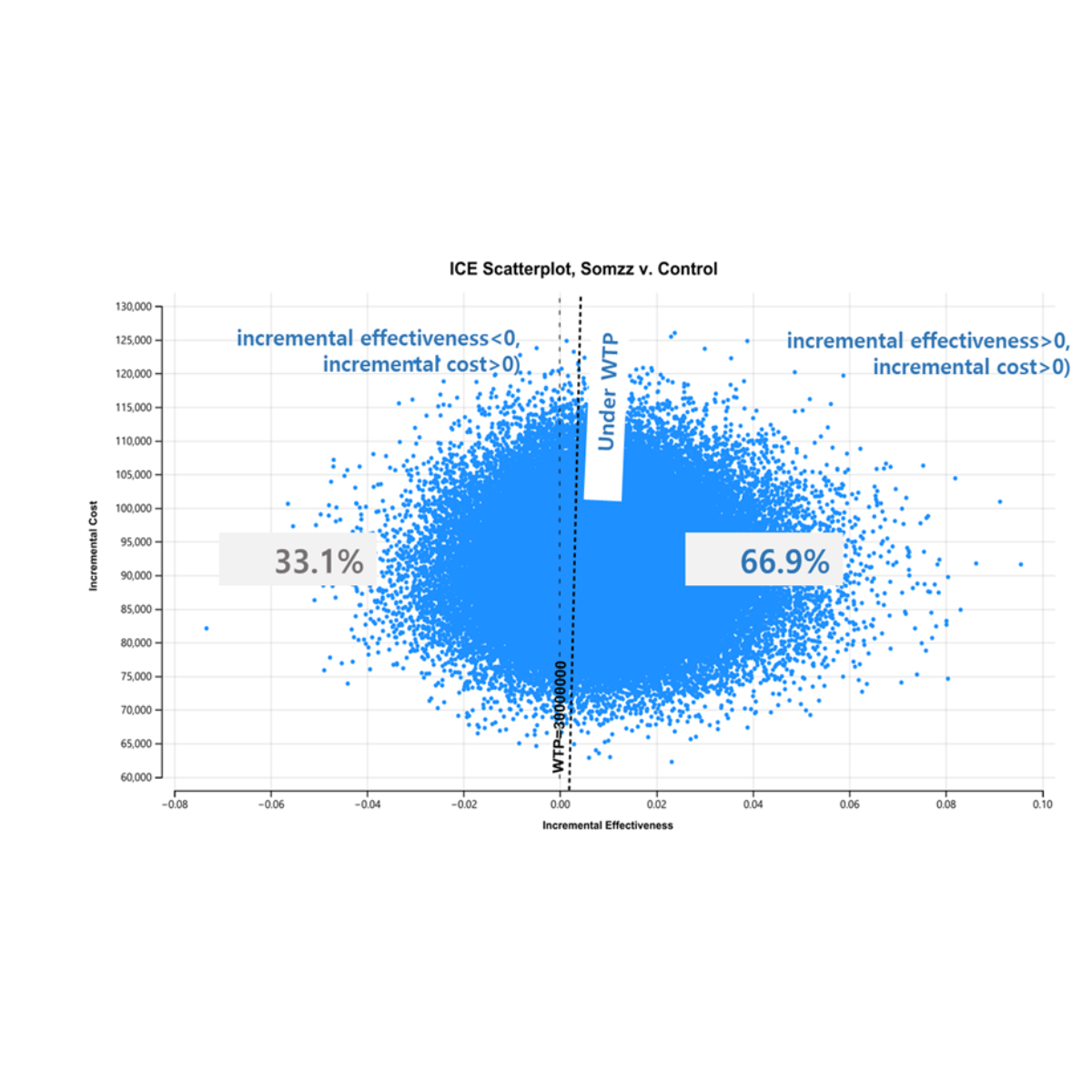

Supplement: Multimedia Appendix 5 [file mhealth_v14i1e71750_app5.png]

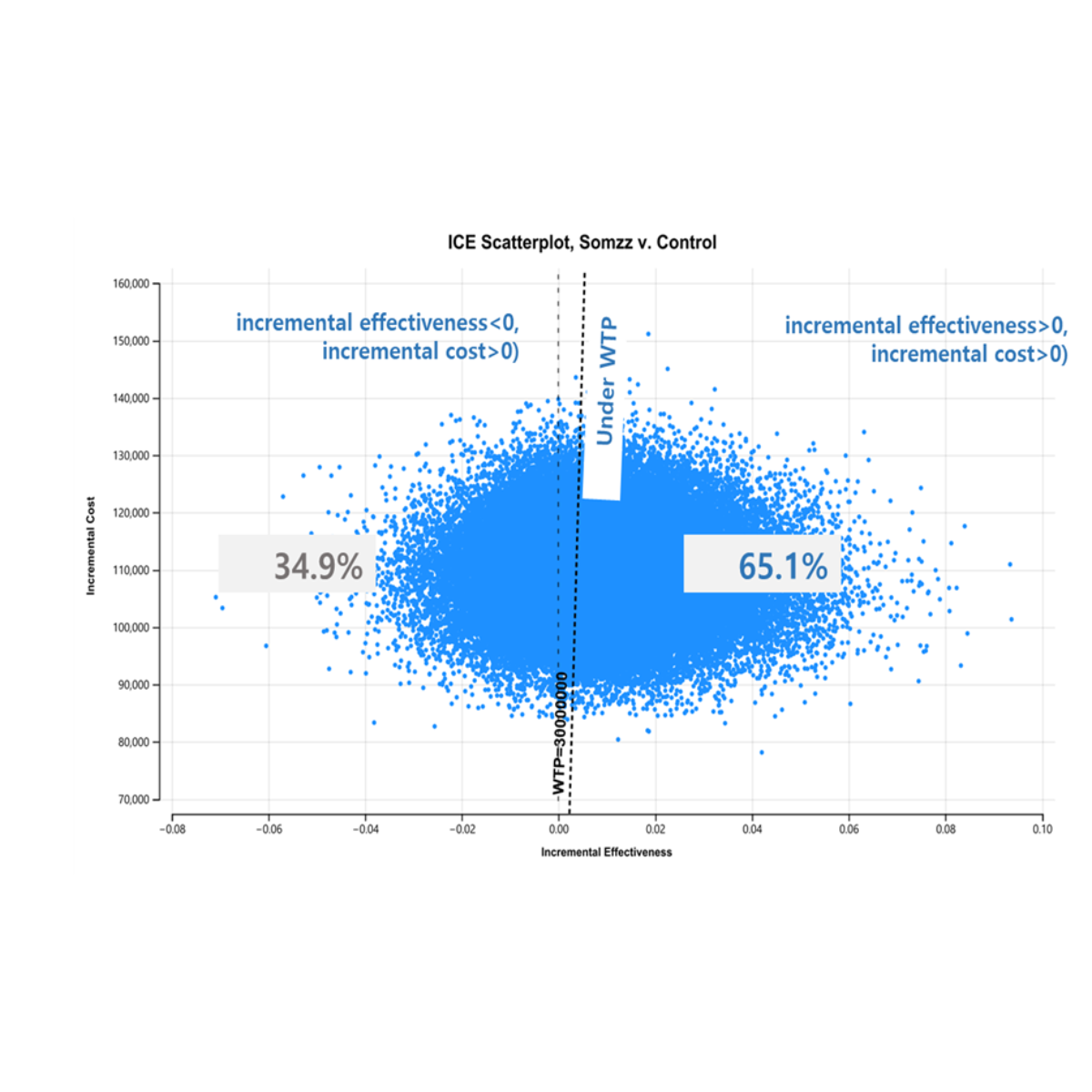

Supplement: Multimedia Appendix 6 [file mhealth_v14i1e71750_app6.png]

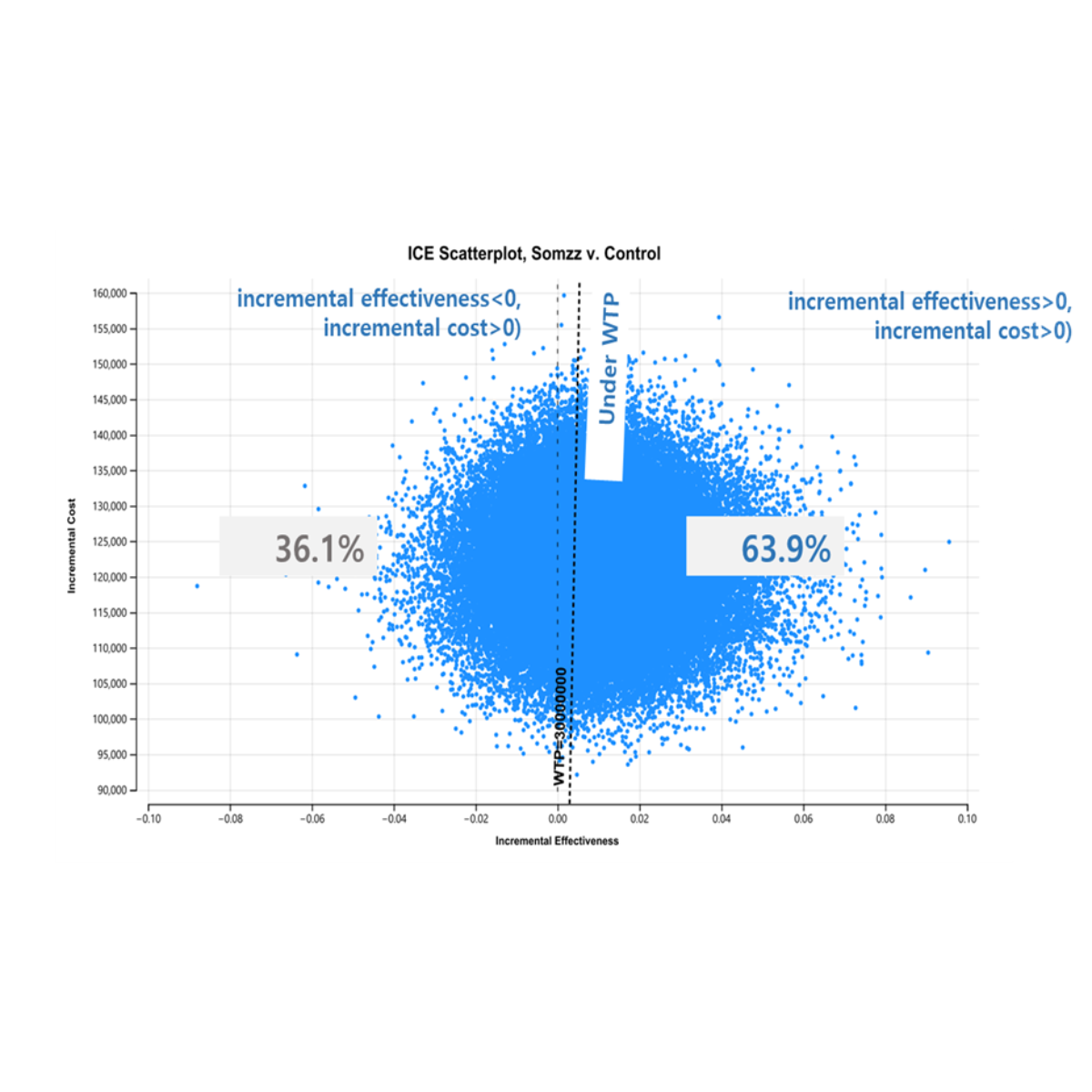

Supplement: Multimedia Appendix 7 [file mhealth_v14i1e71750_app7.png]

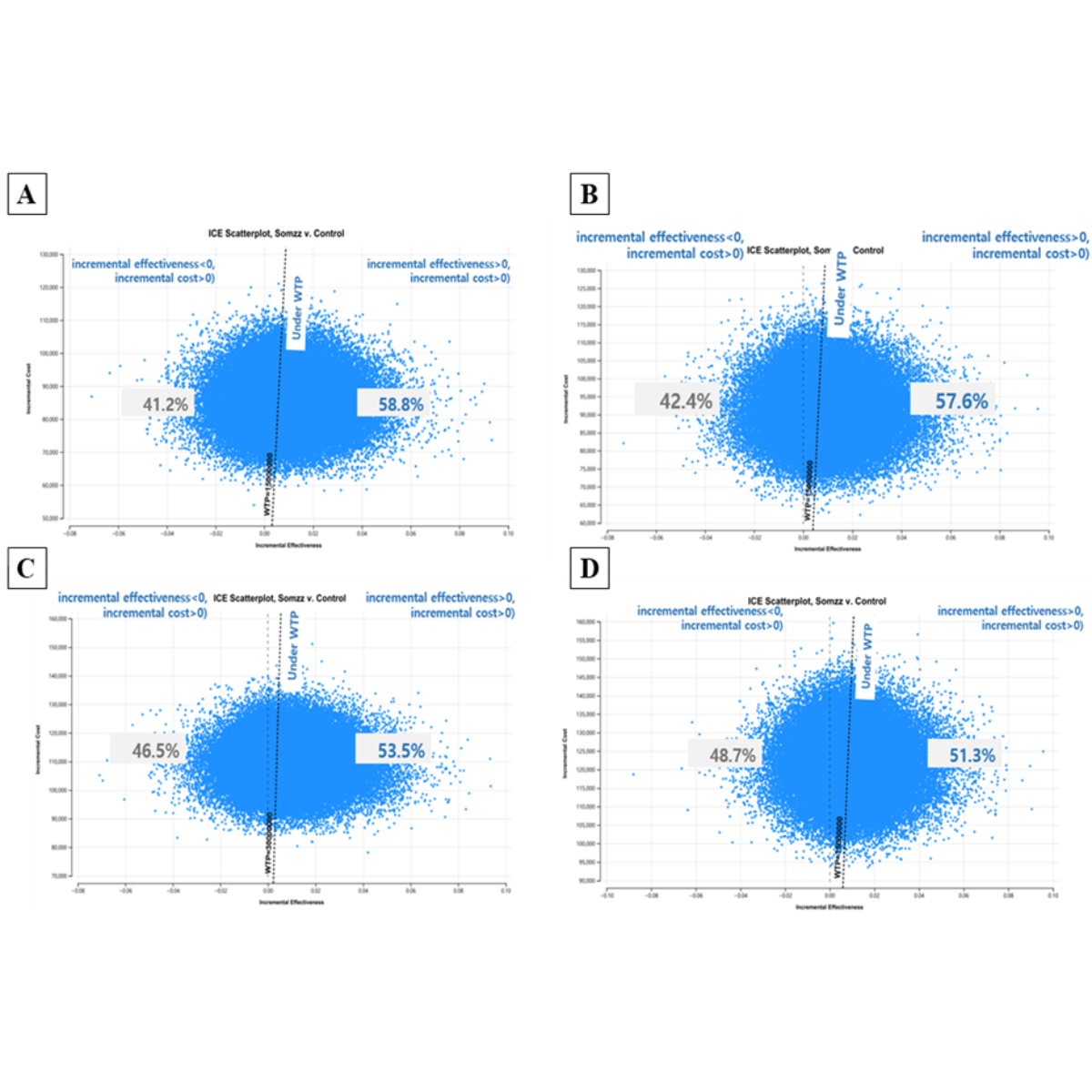

Supplement: Multimedia Appendix 8 [file mhealth_v14i1e71750_app8.png]
